# The influence of wind and basin geometry on surge attenuation along a microtidal channel in the western Baltic Sea

**DOI:** 10.1017/cft.2024.11

**Published:** 2024-11-25

**Authors:** Joshua Kiesel, Arne Knies, Athanasios T. Vafeidis

**Affiliations:** 1 Department of Geography, Kiel University, Kiel, Germany; 2 Institute for Environmental Studies, Vrije Universiteit Amsterdam, Amsterdam, The Netherlands; 3 Institute of Geosciences, Kiel University, Kiel, Germany

**Keywords:** Baltic Sea, hydrodynamic modeling, storm surge, sea-level rise, coastal flooding, surge attenuation, adaptation, managed realignment

## Abstract

The capacity of river mouths to reduce storm surge water levels upstream, referred to as along-estuary attenuation, has been assessed by several studies. The coastal protection function of semi-enclosed water bodies such as lagoons and channels with narrow inlets remains less explored and generalization is hampered by differences in morphology and hydrodynamic forcing. Here we use a hydrodynamic model to investigate surge attenuation along a microtidal channel with a narrow inlet at the Baltic Sea coast of Germany called The Schlei. We quantify the importance of wind and the contribution of the barrier spit system, which is constricting the inlet, to the reduction of water levels at the landward end of the channel. In addition, we explore the role of dikes in the region for the reduction of peak water levels and coastal flooding. We find effective along-channel attenuation inside The Schlei in its current state, which is mostly a result of the channel’s narrows. However, reduction rates decrease under simplified sea-level rise scenarios. Furthermore, along-channel attenuation is highly variable and can change to substantial amplification depending on hydrometeorological forcing. The barrier spit contributes to along-channel attenuation whereas the effect of existing dikes (or their removal) for along-channel attenuation is negligible.

## Impact statement

The southern Baltic Sea coast is facing an increased risk of coastal flooding, which was highlighted by a severe storm surge hitting the southern Danish and German Baltic Sea coasts on October 20 and 21, 2023. One of the regional hotspots of coastal flooding during this event and potentially in the future is the glacially formed microtidal channel known as The Schlei, stretching 43 km from its inlet on the Baltic Sea coast to its western end. The area is of supraregional significance and features a complex geometry consisting of a large barrier spit system constricting the inlet and two narrow passages. The natural coastal protection function of geometrically complex water bodies such as lagoons or channels remains understudied and concerns about the vulnerability of the barrier spit systems to waves, sea-level rise or anthropogenic coastal alterations fosters debates on its future effectiveness in buffering waves and storm surges. Furthermore, it is essential to understand how sea level, meteorological forcing (wind) and adaptation to coastal flooding interact in microtidal channels such as The Schlei. This knowledge can assist coastal managers in developing long-term plans and selecting effective adaptation strategies, not only for The Schlei region, but also for other microtidal coastlines with similar complex water body geometries.

## Introduction

Climate change induced sea-level rise (SLR) will increase the exposure of low-lying coasts to flooding over the course of the century and beyond (Vousdoukas et al., 2017, 2018; Brown et al., 2018). In Europe, the Baltic and the North Sea may experience the highest increase in extreme sea levels until 2100 (Vousdoukas et al., 2017). Among the Baltic Sea nations, Germany is projected to likely suffer severe damages from increased coastal flooding due to climate change, and updates to existing coastal protection measures are required (Vousdoukas et al., 2020; Rutgersson et al., 2022; Kiesel et al., 2023b). This has just recently been demonstrated by a severe storm surge that hit the German and Danish Baltic Sea coasts on October 20 and 21, 2023, when extreme water levels at several locations were roughly equivalent to the 200-year surge water levels calculated in Kiesel et al. (2023b).

**Figure 1.**
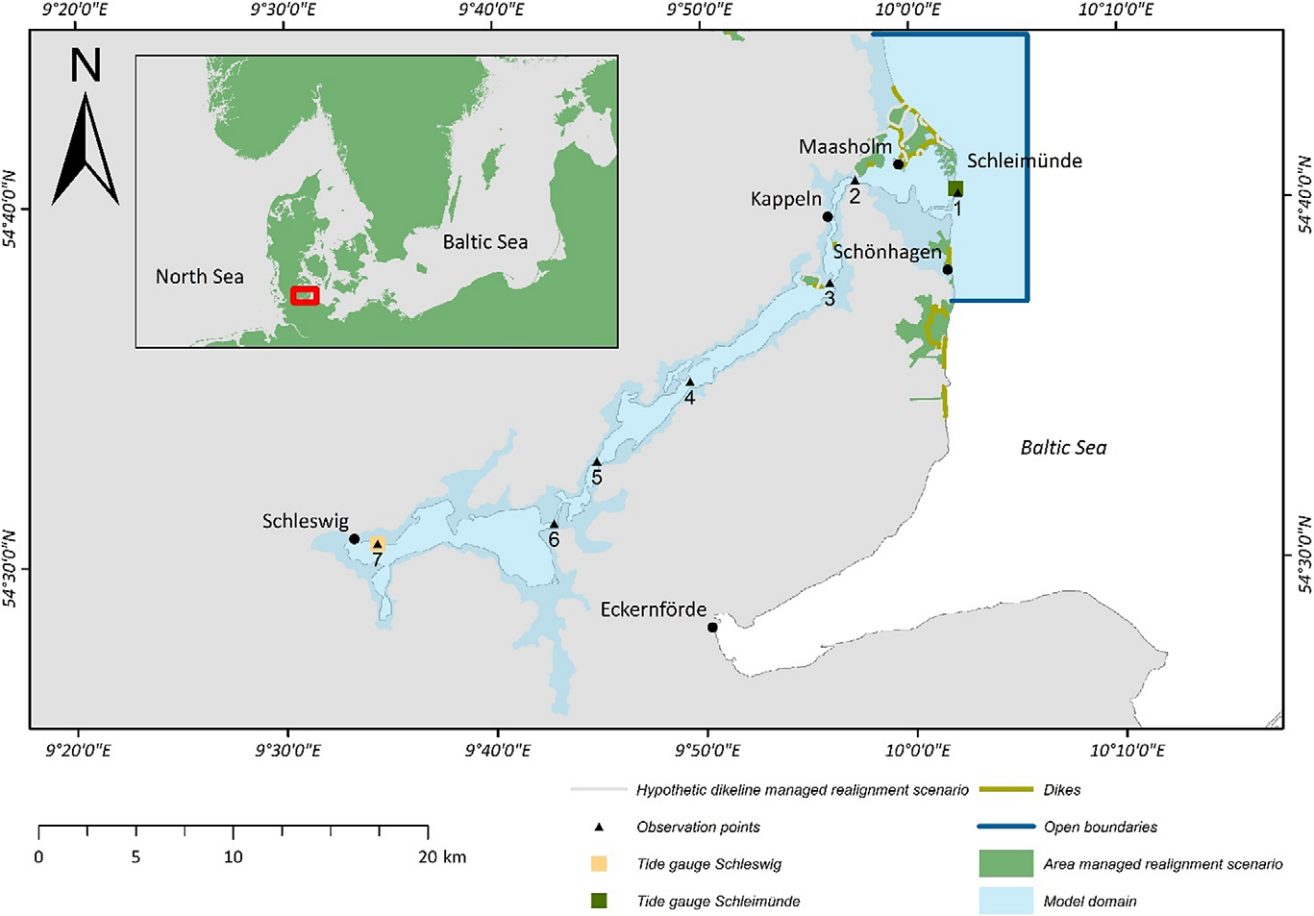
Study area and extent of the hydrodynamic model (blue). Indicated are model observation points and water level gauges. The green areas are identified as potential sites for managed realignment in Kiesel et al. (2023a).

Meteorological forcing generates extreme sea levels in the microtidal Baltic Sea, with the most important contribution coming from storm surges, wind waves and preconditioning. Preconditioning describes increased water levels in the Baltic Sea before the onset of a storm that relates to earlier events, or the system reaction (seiching) (Suursaar et al., 2006; Madsen et al., 2015; Weisse et al., 2021). In the Baltic Sea, storm surges and associated peak water levels can last from a few hours to several days (MacPherson et al., 2019; Wolski and Wiśniewski, 2020). The duration and temporal evolution of storm surges and associated water levels can have important implications for coastal flooding (Kupfer et al., 2024). For example, in the Eckernförde Bay (Figure 1), Höffken et al. (2020) found that differences in flooding extent for a 200-year event and SLR can amount to 20 %, depending on storm surge duration.

River mouths are hotspots of coastal flooding due to their exposure to tides, storm surges, waves from the sea and discharge from the river, and the surrounding flood-prone low-lying land. Moreover, they are often characterized by high population densities and the concentration of assets, which is why they have been in the focus of risk assessment in recent years (Monbaliu et al., 2014; Hanslow et al., 2018; Spicer et al., 2019; Harrison et al., 2022; Kupfer et al., 2022). Funnel-shaped macrotidal estuaries can amplify tides and storm surges, which is dependent on geometric factors such as depth, length and width and their variation (Talke and Jay, 2020). In addition, amplification is exacerbated by human interventions that affect estuarine geometry such as deepening of the channel, straightening, narrowing by land reclamation and constructions (Temmerman et al., 2004, 2013; Familkhalili and Talke, 2016; Talke and Jay, 2020). Therefore, several studies have recently explored the potential of restoring estuarine floodplains and thus the natural water storage capacity, which has often been implemented by means of managed realignment (MR) (Smolders et al., 2015; Fairchild et al., 2021; Weisscher et al., 2022). MR constitutes the breaching or removal of the most seaward dike line in order to allow water exchange, dampening of tidal or flood waves and the (re-)establishment of wetlands such as salt- and freshwater marshes (French, 2006; Esteves, 2014). Increasing the water storage capacity along estuaries can lead to the reduction of storm surge peak water levels upstream, which has been referred to as *along-estuary attenuation* (Smolders et al., 2015). Along-estuary attenuation is particularly effective when wetlands are restored upstream of the estuary domain (Smolders et al., 2015; Fairchild et al., 2021).

Other than the funnel-shaped estuaries, complex or concave coastlines such as bights and lagoons or microtidal river mouths with narrow inlets may feature other combinations of forcing conditions (microtidal, storm dominant, seiche) and characteristics with regard to attenuation and amplification of peak water levels (Llebot et al., 2014; Li et al., 2018; Ferrarin et al., 2022). It is well established that inlets reduce long wave amplitudes and act as a low-pass filter decreasing short period waves. Also, any direct or indirect changes to inlet width and depth affect the degree of attenuation (Talke and Jay, 2020). Consequently, system dynamics depend on the complex interplay of morphology, discharge and hydrodynamic forcing conditions. The western Baltic Sea coast features microtidal inlets that connect fjords or subglacial meltwater channels with the sea. Due to their sheltered location and navigability, major cities have developed such as Odense or Roskilde in Denmark and Schleswig, at the western end of The Schlei.

The Schlei is a microtidal channel that is located in the northeast of the German federal state of Schleswig-Holstein (Figure 1); it is a 43 km long and narrow water body that is connected with the sea via a 100 m wide inlet. The inlet is characterized by a large barrier spit system, limiting water exchange with the sea and providing natural coastal protection against waves. In the face of SLR and human interventions, debates about the future persistence of sand barrier systems, overwash and breaching are on the rise (Carrasco et al., 2016), evoking questions as to how these features are and will contribute to coastal protection in the form of surge attenuation and the reduction of flooding extents. Due to the microtidal regime and limited freshwater input (Seiß, 2014), water level fluctuations inside The Schlei are mainly driven by changes in water level of the adjacent Baltic Sea (Schwarzer et al., 2019).

It is not known whether and how the established concept of along-estuary attenuation (Smolders et al., 2015; Fairchild et al., 2021) may be applicable to semi-enclosed microtidal coastal waterbodies such as The Schlei (hereafter referred to as along-channel attenuation) and which factors drive the system’s internal peak water level variability. For instance, research is needed to investigate the effects of wind stress on the dynamics of peak water levels along estuaries and microtidal channels alike (Talke and Jay, 2020). Furthermore, anthropogenic alterations to the system, such as the role of potential future adaptation measures including both conventional and nature-based solutions and the importance of the narrow inlet for peak water level dynamics should be investigated.

In order to understand The Schlei’s extreme event hydrodynamics under current and projected scenarios, we use a hydrodynamic model based on the Delft3D-Flow modeling system (Delft Hydraulics, 2003). We simulate two storm surges with different characteristics in terms of peak-water level, duration and wind pattern that occurred in the study region. The first occurred between March 16 and 18, 2018 and the second on January 2, 2019. The 2019 event was caused by a cyclone (German name: Zeetje) shifting southeast from Lofoten Islands (980 hPa) to Estonia, in combination with a high-pressure zone over the English Channel leading to winds in the southwestern Baltic Sea of 5–6 Beaufort from southwest, then 6–7 from west and later 7–8 from northwest (Perlet, 2019). At first, this led to falling and subsequently rising water levels, the maximum being recorded at the southernmost point of the Baltic Sea in Wismar (1.93 m). During the 2018 event, winds were caused by an anticyclone (German name: Irenäus) shifting west from Norway (1032 hPa) to the Faroe Islands and leading to 7 Beaufort easterly winds (Perlet, 2018). At the German Baltic Sea coast, the highest water levels were recorded in Schleswig, located at the western end of The Schlei (1.41 m).

We combine the 2019 event with three regional SLR projections from the 6th assessment report of the Intergovernmental Panel on Climate Change (IPCC) (Fox-Kemper et al., 2021). We select two scenarios for the year 2100 corresponding to medium confidence (SSP1-2.6 50th percentile (MSL+0.5 m) and SSP5-8.5 50th percentile (MSL+0.85 m), hereafter referred to as SSP1-2.6 medium and SSP5-8.5 medium) and one scenario as a high-end projection (low confidence) (SSP5-8.5 95th percentile (MSL+2.15), hereafter referred to as SSP5-8.5 high). We have selected the above scenarios to cover the range of uncertainty of plausible futures and also included a high-end projection as these are particularly relevant for coastal management (Hinkel et al., 2019; Stammer et al., 2019; van de Wal et al., 2022). We simplify these scenarios in that the unknown geomorphic response of the natural system to future SLR is not accounted for.

This study has two main objectives: First, it aims to enhance the system understanding of fjord-type microtidal channels using the example of The Schlei, by quantifying the importance of sea-level rise, wind and the contribution of the barriers spit system to along-channel attenuation. Second, it focuses on human adaptations of the system by addressing the function and role of existing dikes in the study region in terms of along-channel attenuation and coastal flooding. We study the function of the dikes by raising their height and implementing hypothetical MR sites where physically plausible.

## Study area and methods

### Study area

As part of the German Baltic Sea coast, The Schlei was morphologically shaped during and after the last glaciation of Northern Europe (Weichselian Glaciation) and is composed of glacial drift material and basin deposits (Sterr, 2008). The Schlei can be described as a 43 km long chain of three basins that are connected by three narrow passages (Seiß, 2014). The channel is a result of subglacial meltwater erosion, and its width in the basins is mostly between one and two kilometers but narrows down to ca. 100 m at the three narrow passages. Average water depths vary between 2.5 m and 3 m, and maximum depths of 16 m NHN (local reference datum, where 0 m approximates to mean sea level) are reached at The Schlei’s narrows (Schwarzer et al., 2019).

The water exchange with the Baltic Sea is nowadays limited by the narrow, 100 m wide inlet breaching the Schleimünde sand spit barrier system (Figure 1). The development of the barrier has not yet been entirely reconstructed, but it likely initiated between 4000- and 2000-years BP. In those times two spits grew from north and south and merged in the 1960s. Since the late 18th century an artificial channel is maintained in order to allow for water exchange and the passage of ships (Schwarzer et al., 2019). The opening is now protected by groins to the north and south of the inlet.

The microtidal regime of the Western Baltic Sea features tidal ranges between 0.1 and 0.2 m (Sterr, 2008). The 30- and 200-year return water levels are 1.69 ± 0.15 m and 1.98 ± 0.26 m for Schleimünde and 1.91 ± 0.28 m and 2.44 ± 0.65 m for Schleswig, respectively (Kiesel et al., 2023b). SLR rates in the Baltic Sea are broadly consistent with the global average of 3–4 mm yr^−1^ (Meier et al., 2022). However, relative SLR is variable. Caused by the Fennoscandian ice shield of the last glaciation, isostatic adjustment remains to uplift the shores of the northern Baltic Sea coast, while southern areas, including parts of the German Baltic Sea coast, are subsiding (Richter et al., 2012; Dangendorf et al., 2022).

The western end of The Schlei marks the narrowest part of the Jutland peninsula, providing proximity to the North Sea. Therefore, and because of its sheltered location, the Viking town Hedeby (ca 2.5 km south of Schleswig) prospered in the 9th century as a leading trade center of the Danish kingdom until its final destruction in AD 1066 (Hilberg, 2009). In 2018, Hedeby gained supra-regional significance when it was designated as a UNESCO world heritage site (Khamnueva‐Wendt et al., 2020).

### Data

To set up the hydrodynamic model, we compiled data on topography, bathymetry, water levels from local tide gauges, land cover and dikes. Most of the data, including topography, bathymetry, water levels and dikes, were provided by state authorities while land cover was obtained from Corine (© European Union, Copernicus Land Monitoring Service 2018, European Environment Agency [EEA]). Information about resolutions, accuracy and sources of the data are summarized in Supplementary Table S1.

### Model setup

We used the hydrodynamic modeling system Delft3D-FLOW, which we applied in horizontally two-dimensional (2DH) mode. The model solves the depth-averaged Shallow-Water Equations on a discrete curvi-linear grid (Delft Hydraulics, 2003). The modeling system simulates time-varying flow conditions including velocities, water levels and sediment transport over a two- (or three-) dimensional finite difference grid. Delft3D is well suited for riverine, estuarine or coastal applications and has been used in several studies in the past, including the Baltic Sea (Lesser et al., 2004; Temmerman et al., 2005; Lyddon et al., 2018; Kumbier et al., 2019; Höffken et al., 2020).

We used a Cartesian grid with 50 m resolution to simulate two-dimensional (depth-averaged) flow. The perimeter of the model domain was defined by including all areas below 5 m elevation that were hydrologically connected to The Schlei. To identify those areas, we used a 1 m Light Detection and Ranging (LiDAR) derived digital elevation model (DEM) (Supplementary Table S1).

In order to ensure unobstructed flow through The Schlei’s narrow passages, we positioned the computational grid in a way that locations at which depth was specified in the model matches the locations of datapoints in the bathymetry dataset, so that no interpolation was required. Further manual adjustments had to be done to correctly represent the hydraulic connections of semi-enclosed lagoons in the study region (namely the Lindauer and Ornumer Noor) and to ensure the correct elevation of the harbor wall in Olpenitz port (south of Schleimünde) and the beach ridge along the barrier spit system.

Land elevation was compiled from 1 and 10 m LiDAR DEMs (Supplementary Table S1). The preparation of the DEM followed the procedure described in Kiesel et al. (2023b). We first resampled the 10 m LiDAR data to 50 m to achieve consistency with the bathymetry data. We incorporated the dike heights into the 50 m elevation dataset by first extracting the dike crests of the 1 m DEM within a 100 m buffer around each dike line (Supplementary Table S1). Second, we aggregated these data to 50 m using maximum values and combined it with the 50 m elevation data. As for the bathymetry data, we overlaid the elevation with the computational grid, so that grid interpolation did not change the adjusted elevation data.

We applied a spatially varying surface roughness within the model domain based on Manning’s *n* coefficients (Garzon and Ferreira, 2016; Lopes et al., 2022). The coefficients were applied based on land cover classes present in the publicly available Corine dataset (European Union, Copernicus Land Monitoring Service 2018, European Environment Agency [EEA]) that we reclassified according to Supplementary Table S2. The Corine land cover data includes water surface areas. The final combination of coefficients and the different setups applied during model calibration can be found in section “Model calibration and validation.”

The model was forced with timeseries of water levels and wind. Water levels were prescribed at the eastern open boundary, while the two boundaries perpendicular to the coast were not forced (Figure 1). At the eastern open boundary, time varying flow conditions were initiated with tide gauge data from Schleimünde (provided by the Federal Waterways and Shipping Administration (WSV)). Hourly data on wind speed and direction was provided by the German weather service (DWD) for the station in Schönhagen (Figure 1). We applied a spatially uniform wind field throughout the model domain and the time step of the model simulations was 1.2 seconds. The three SLR scenarios were added linearly to the water levels at the eastern open boundary. The model was run without a specified spin-up period.

### Model calibration and validation

The model has been applied largely uncalibrated. Only the sensitivity of the model to variations in surface roughness was tested according to different setups of Manning’ *n* coefficients (see Supplementary Table S3) for the storm surge of January 2, 2019. We tested four setups of coefficients based on Kiesel et al. (2023b) and calculated the difference between modeled and measured water levels for the tide gauge in Schleswig (Figure 1). Root mean square errors (RMSE) for a model setup with high, low and moderate Manning´s coefficients varied between 0.3, 0.09 and 0.18 m, respectively. Applying the setup Moderate 2 (Supplementary Table S3) resulted in a RMSE of 0.08 m. In this setup, we have only reduced the roughness coefficient for inland water bodies (i.e., The Schlei) as compared to the Moderate setup (bold number in Supplementary Table S3). The final model setup used a Manning’s *n* coefficients based on the Moderate 2 configuration.

**Figure 2.**
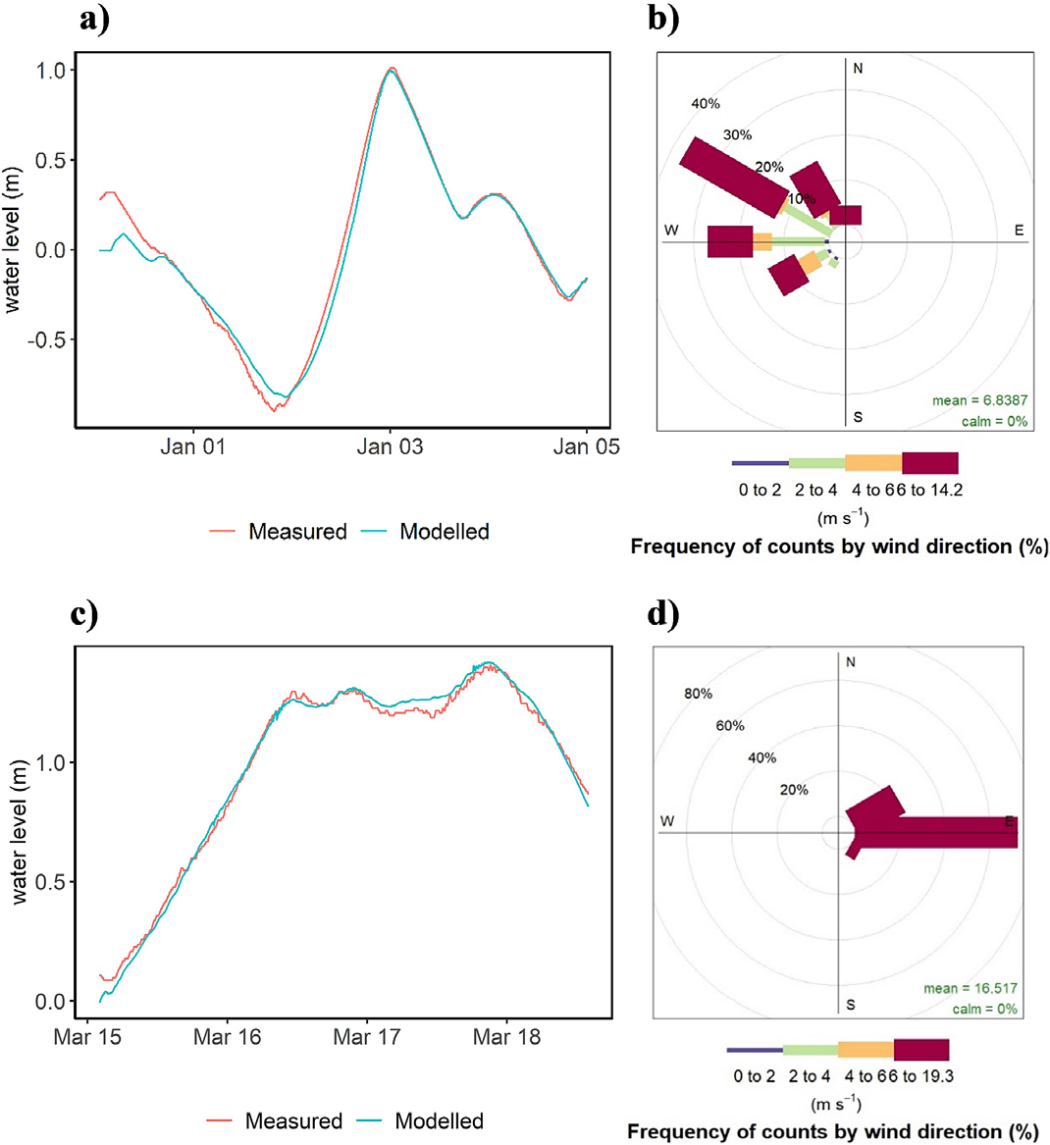
Observed and modeled water level timeseries at the tide gauge Schleswig for the storm surges of a) January 2019 and c) March 2018. Panels b) and d) depict the corresponding wind conditions for the respective periods at Schönhagen (see Figure 1). The colored bars indicate wind speed and the direction from which the wind is coming.

We validated the model using observed water levels at the tide gauge in Schleswig for the storm surge that occurred in mid-March 2018 (Figure 2c,d) (simulation period 2018-03-15–2018-03-18). We compared modeled and measured water levels using RMSE, mean absolute error (MAE) and the difference in peak water level. We found that the model was well capable of reproducing measured water levels in Schleswig, as expressed in a RMSE of 0.04 m, a MAE of 0.03 m and a difference in peak water level of 0.01 m (Figure 2c and Supplementary Figure S1).

Furthermore, we validated the model by means of flooding extents using Sentinel-1 SAR imagery acquired at 17:08 UTC on January 2, 2019, which is 3 hours after the measured peak of the surge in Schleimuende. The processing of the SAR imagery is described in Kiesel et al. (2023b). We find that modeled and measured flooding extents are in good agreement even though the SAR imagery indicates a lower flooding extent (5.26 km²) than simulated in this study (6.32 km²). As compared to the SAR imagery, we estimated that 60% of the cells were predicted correctly, 42% were overpredicted and 40% were missed (see Supplementary Figure S2).

### Implementation of dike height increases, MR and flood protection function of the barrier spit system

#### Flood protection function of the barrier

In order to investigate the role of the barrier spit system on flooding extents and along-channel attenuation, we created an artificial DEM-bathymetry dataset (see also section “Model setup”). We “removed” the barrier by delineating an interpolation area in the DEM, which covers the area from just seaward of the barrier and extends inland to observation point 2 (Figure 1) (interpolation area is depicted in Supplementary Figure S3). We then removed all cells within the interpolation area that were higher than −3 m (e.g., the barrier spit system) to ensure that new values were only interpolated for areas above −3 m. Next, we interpolated new elevation values using the Kriging algorithm in ArcMap (version 10.8.1). In a final step, we combined the interpolated bathymetry values with the original bathymetry-elevation dataset (see section “Model setup”). Finally, Manning’s *n* coefficients for cells that previously represented the barrier were changed to represent open water (0.02, see Supplementary Table S3).

#### Dike height increase

The General Plan for Coastal Protection of Schleswig-Holstein introduces the so-called climate dikes, which are dikes constructed with a wider base, allowing for height increases of up to 1.5 m to account for future SLR (Melund, 2022). Dike height increases in this study were incorporated according to the methodology described in Kiesel et al. (2023a). In this approach, 1.5 m was added to all cells in the model DEM that were higher than 0.5 m elevation in a 100 m buffer around each dike line. The buffer was used to account for inaccuracies between the dike line and the associated raster cells. The elevation threshold of 0.5 m was used to ensure that only dike elevations are increased and not low-lying land adjacent to the actual dike. Since storm surge water levels overtop the dikes near The Schlei’s inlet only under the highest SLR scenario (SSP5-8.5 high), we run only this scenario with increased dike heights.

#### Increasing The Schlei’s water storage capacity by implementing hypothetical MR sites

We used the perimeters of potential MR sites (see Figure 1), identified in Kiesel et al. (2023a), to investigate the impacts of dikes for coastal flooding extents and along-channel attenuation. The used MR detection algorithm consists of the following steps: First, the maximum inland extent of potential MR sites was defined to be 1,800 m. This distance was measured as the distance between the coastline and the hypothetical new, landward dike line of each MR site and corresponds to the average width of the ten largest MR sites in Europe listed in the Online Marine Registry (OMReg) database as of July 2022 (ABPmer, 2022). Second, segmented buffer zones were implemented in 100 m horizontal and 100 m perpendicular increments along the length of the original dikes. The vertical incrementation was repeated until the maximum inland distance was reached (1,800 m). Third, potential segments for MR implementation were identified when they were on land below 10 m elevation and free from any kind of critical infrastructure such as roads, railways and built-up areas. Built-up areas were identified according to the GHS Built Up Layer (Pesaresi et al., 2015) and roads and railways were taken from the Open Street Map (OSM) Road and Railway layer. Last, we only considered those potential MR sites that were larger than 1 ha.

MR sites were incorporated into the model by adjusting the DEM and the surface roughness inside the potential MR sites. The implementation followed three main criteria: (1) The standard of protection (height of the dike) of the hypothetical new, landward dike was similar to the height of the breached original dike plus 1.5 m to account for future SLR (Melund, 2022). (2) The original seaward dike line needs to be breached in order to allow flooding of the adjacent low-lying areas. (3) Parts of the old (original) dike line that are located inside the potential MR areas should be removed. Finally, surface roughness coefficients were adjusted for the potential MR areas, so that they represent the drag induced by wetlands that are anticipated to be restored by means of MR. For further details regarding the detection and implementation of hypothetical MR sites, we refer the reader to the original publication (Kiesel et al., 2023a).

## Results

### System understanding

#### Wind as a major driver of along-channel attenuation

**Figure 3.**
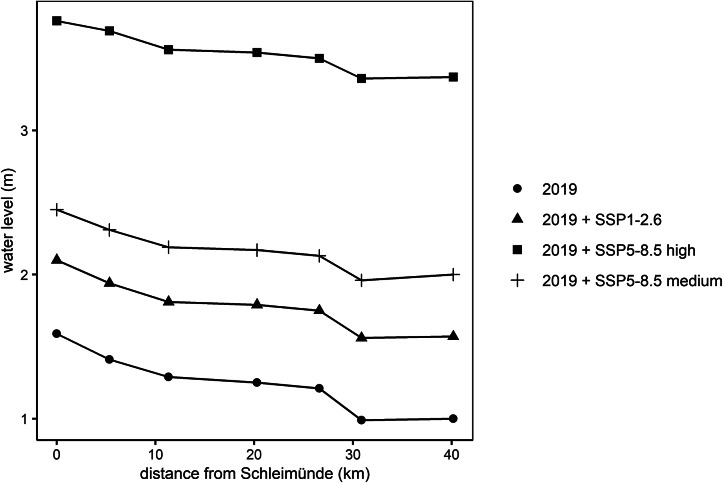
Peak water levels for model observation points between Schleimünde (km 0) and Schleswig (km 40) for the storm surge of January 2, 2019 and three SLR scenarios.

For the storm surge that occurred on January 2, 2019, we find that The Schlei effectively reduces peak water levels between Schleimünde and Schleswig, but the effectiveness is reduced under higher sea levels (Figure 3). Without SLR, the peak water level is reduced from 1.59 m at the inlet to 1 m in Schleswig (37%). For the highest SLR-scenario modeled in this study (SSP5-8.5 high), peak water levels are reduced from 3.76 m at the inlet to 3.37 m near Schleswig (10%). Our results further clarify that the modeled attenuation is primarily occurring at The Schlei’s narrow passages between observation points one and two (the inlet), two and three and five and six (from left to right in Figure 3, see also Figure 1).

**Figure 4.**
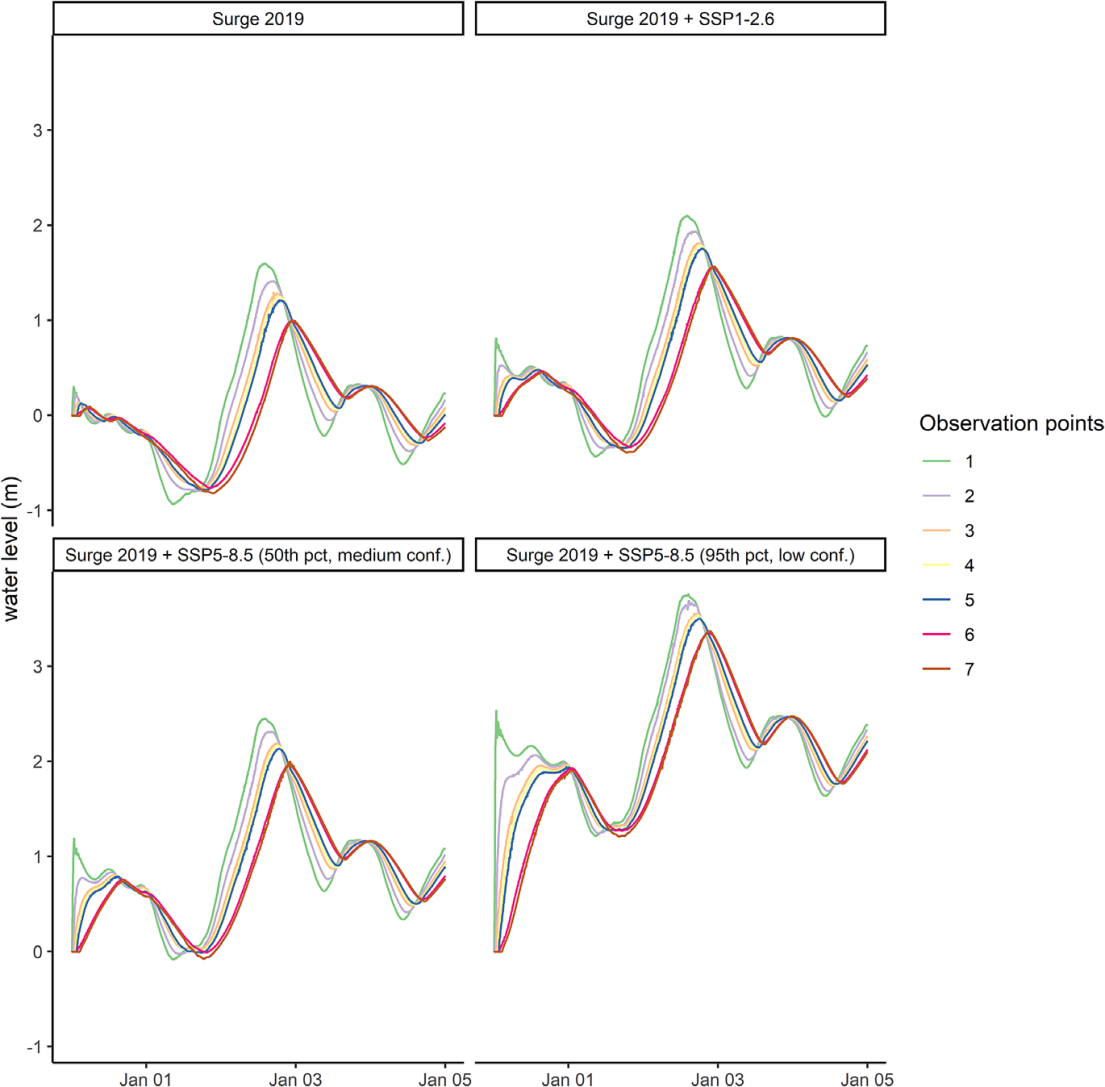
Water level timeseries for the storm surge of January 2, 2019 and three SLR scenarios. Observation points correspond to Figure 1.

We further show that not only peak water levels are reduced, but also the timing of the peak water level in Schleswig is considerably delayed compared to Schleimuende (Figure 4). For the storm surge of 2019 without SLR, the peak of the surge arrived 9 hours and 5 minutes later in Schleswig (observation point 7) as compared to Schleimuende (observation point 1). This delay is reduced by two hours under the highest SLR scenario simulated for this study (SSP5-8.5 high) to 7 hours and 5 minutes.

**Figure 5.**
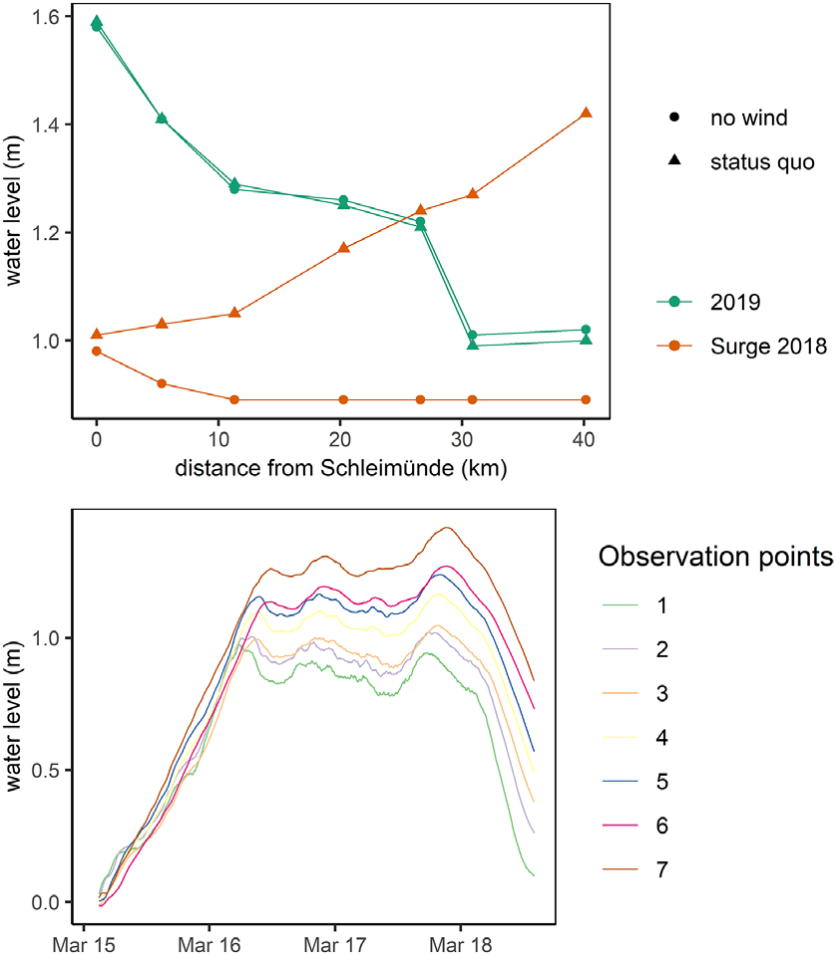
Top: Peak water levels for model observation points during the 2019 and 2018 storm surges with and without wind. Bottom: Water level timeseries for all observation points along The Schlei during the 2018 storm surge. Observation points correspond to Figure 1.

Differences in along-channel attenuation between the 2019 and 2018 storm surges suggest that wind direction during the surge has a major influence. While the wind direction during the simulation period of the 2019 storm surge was predominantly from west and northwest, the 2018 storm surge was characterized by easterly and northeasterly winds (Figure 2d). The along-channel attenuation between the two events is equally opposite. We modeled an overall attenuation between Schleimuende and Schleswig of 59 cm (37%) for the 2019 event, while the 2018 surge has resulted in an amplification of peak water levels by 41 cm (41%) (1.01 m in Schleimünde and 1.42 m in Schleswig) (Figure 5). While the exclusion of wind in the 2019 storm surge has only minor effects on along-channel attenuation, the 2018 surge changes from amplification to attenuation (9 cm) (Figure 5). Apart from differences in peak water levels at The Schlei’s inlet (1.59 m for the 2019 surge and 1.01 m for 2018), the 2018 surge is considerably longer, with water levels around 1 m in Schleimünde lasting for more than 2 days (Figures 2 and 5).

#### The contribution of the barrier spit system to along-channel attenuation and flooding extent

The barrier spit contributes to along-channel attenuation, as peak water levels in Schleswig (observation point 7) during the 2019 surge are 9% (9 cm) higher when it is removed in the model (as compared to status quo). At observation point two (right behind the inlet), the presence of the barrier reduces peak water levels by 13 cm (1.41 m with and 1.54 m without barrier). For the selected SLR scenarios the influence of the barrier on along-channel attenuation is reduced. Under the highest SLR scenario (SSP5-8.5 high), the peak water levels in Schleswig and at observation point 2 are only 4 cm and 5 cm higher, respectively, when the barrier is removed (see also Supplementary Figure S4). During the 2019 surge, the barrier spit system has furthermore delayed the arrival of the peak of the surge in Schleswig by 45 minutes. Specifically, the counter-factual removal of the barrier results in the arrival of the peak of the surge in Schleswig eight hours and 20 minutes later as compared to Schleimünde (vs. 9 hours and 5 minutes with barrier in place, see section “Wind as a major driver of along-channel attenuation”). As for the status quo model, the delay is reduced when sea levels rise. A model run without barrier spit system and under the highest SLR scenario results in the surge peaking after 7 hours and 10 minutes at the western end of The Schlei. This is 5 minutes later than with the barrier in place, suggesting the reduced influence of the barrier spit system on The Schlei’s peak water level dynamics under high SLR scenarios.

The barrier spit system plays only a minor role in reducing the flooding extent along The Schlei. Depending on the SLR scenario, the removal of the barrier results in increases of 1% and 6% in flooding extent, for SSP5-8.5 high and SSP1-2.6, respectively (Table 1). For this comparison, the flooded area on the sandspit in the status quo model runs was excluded (see Table 1).

**Table 1.** Flooding extents for the different SLR scenarios, the counterfactual barrier spit system removal and dike adaptation scenarios

	Surge 2019 (ha)	SSP1-2.6 (ha)	SSP5-8.5 medium (ha)	SSP5-8.5 high (ha)
Status Quo/excluding flooded barrier	709.25/646.25	986.25/896.75	1166/1068.25	2981.5/2876.25
No barrier	676.5	953.25	1105.5	2897
Dike height increase	NA	NA	NA	2600.5
Managed realignment	934	1201.75	1363.75	2765

NA, not assessed.

### Flooding extents under SLR scenarios and landscape changes

Our results show that the exposure of The Schlei’s coast to flooding may increase under SLR, while the here tested changes to existing coastal protection measures may not compensate for this increase. Maximum flooding extent varies between 709 ha for the 2019 storm surge (status quo) and 2981.5 ha for the 2019 surge under SSP5-8.5 (high) (Table 1).

The larger flooding extents for the 2019 storm surge and the SLR scenarios SSP1-2.6 and SSP5-8.5 medium, for model runs with hypothetic MR sites, are a consequence of the larger area deliberately exposed to coastal flooding in the model (Table 1 and Supplementary Figure S5). Only in the highest SLR scenario is the flooding extent of the model run with MR smaller than in the status quo, which is explained by the associated dike height increases of the new landward MR dikes in that scenario (see section “Increasing The Schlei’s water storage capacity by implementing hypothetical managed realignment sites”). Contrary to changes in flooding extents, the implementation of hypothetic MR sites does not affect along-channel attenuation (Supplementary Figure S4). Across both storm surges (2019 and 2018) and all SLR scenarios, differences in peak water level for all observation points do not exceed 2 cm, where MR either leads to similar or slightly lower water levels.

Without upgraded adaptation, current dikes can be overtopped under the highest SLR scenario simulated for this study. In such a case, dike height increases by 1.5 m could constitute an effective measure to reduce the flooding extent adjacent to The Schlei’s inlet (Table 1 and Supplementary Figure S5). The overall reduction in The Schlei´s flooding extent from dike height increases for this storm surge scenario amounts to 13%.

## Discussion

### Along-channel attenuation can switch to amplification depending on hydrometeorological forcing

As an analogue to along-estuary attenuation (Smolders et al., 2015), we use the term along-channel attenuation to describe the reduction of peak water levels along The Schlei. We note that the attenuation of peak water levels along The Schlei is not linear, as most attenuation is occurring at The Schlei’s narrow passages (Figure 3). The non-linearity in the provision of the natural coastal protection function of wetlands is well known (Koch et al., 2009), suggesting that the common simplification of extrapolating water level reduction rates in cm km^−1^ can be misleading (Grant and Cooker, 2023). This non-linearity is illustrated by the strong spatiotemporal variability in along-channel attenuation rates, which can even turn into amplification depending on hydrometeorological forcing. In this study, we found differences varying between 59 cm (37%) peak water level reduction and 41 cm (41%) of peak water level amplification, depending on the simulated storm surge (Figure 5). In other words, while the peak of the 2019 surge at The Schlei’s inlet was 59 cm higher than the peak of the 2018 surge (1.59 vs. 1 m, respectively), water levels at the western end of the channel in Schleswig were 41 cm higher during the 2018 surge (1.42 vs. 1.01 m).

Similar to funnel-shaped estuaries or geometrically more complex lagoon systems, the coastal protection function of The Schlei is highly variable and flood risk can actually increase landwards due to rising peak water levels. The amplification of water levels along tidal estuaries and lagoons has been observed previously and was related to the funneling of flows during storm surges, the influence of SLR, embankment construction and reduced dampening at the inlet (Picado et al., 2013; Temmerman et al., 2013; Lyddon et al., 2018; Pinheiro et al., 2020; Pareja‐Roman et al., 2023; Pein et al., 2023). The example of The Schlei further suggests that the combination of basin geometry and meteorological conditions may constitute particularly favorable conditions to result in the amplification of peak water levels.

Long residence time of extreme sea levels (i.e., surge duration) constitutes another mechanism favoring the amplification of peak water levels and affecting coastal flooding (Kupfer et al., 2024). Such residence times can be increased by basin geometry as has been described for Baltic Sea lagoons (so-called Bodden). In coastal estuaries, lagoons or channels with narrow inlets, the restricted flow to the open Baltic Sea results in more stable water levels (MacPherson et al., 2019), as small inlets dampen the magnitude of long waves such as tides or surges (Aretxabaleta et al., 2017). Previous research on the natural coastal protection function of wetlands has shown that short events are more effectively attenuated (Grant and Cooker, 2023) and Resio and Westerink (2008) clarified that amplification can occur when the surge duration is long compared to the time it takes to fill the storage area. In the microtidal Baltic Sea, storm surges can persist for days (MacPherson et al., 2019), providing sufficient time to fill up large storage areas. However, our results clearly demonstrate that even the storm surge of 2018, where peak water levels of approximately 1 m above the local reference datum lasted for more than two days, was attenuated when the effects of wind were excluded (Figure 5).

### SLR will disproportionally increase peak water levels inside The Schlei as along-channel attenuation is reduced

Our findings are in line with Lorenz et al. (2023) who suggest that peak water levels inside semi-enclosed water bodies such as lagoons and inlets may rise disproportionally (non-linearly) under the influence of SLR. In this study, the selected SLR scenarios confirm that peak water levels can increase disproportionally in the inner Schlei, as the natural buffer in the form of along-channel attenuation is reduced when inundation depth increases (Figures 3 and 4). While our study points toward the influence of SLR on non-linear peak water level increases, previous assessments demonstrated similar effects as a consequence of dredging thus deepening of the main estuarine channel. Increased channel depth means that friction induced by bottom roughness is reduced, in turn leading to reduced energy dissipation (Familkhalili and Talke, 2016; Talke and Jay, 2020). This has implications for the future management and planning of coastal lagoons, channels, and estuaries with respect to SLR and the natural coastal protection functions these systems provide. In addition, the disproportional rise in peak water levels with SLR is due to increased water volumes entering the system through the narrow passages and the inlet, which is a result of the larger cross-sectional area that water can flow through when sea levels rise (Lorenz et al., 2023). There is consequently a growing interest in how inlets formed by spits and barriers develop under changing hydrodynamic regimes, as they contribute to controlling the water volumes that enter the system. It is noted though, that most studies suggest barriers to be resilient to SLR due to landward barrier retreat through continuous migration, and a gradual change in basin hypsometry during the retreat process (Carrasco et al., 2016).

We find that the barrier in Schleimünde, which is one of the three narrow passages of The Schlei, is partly contributing to the overall reduction in peak water levels between the inlet and Schleswig. We must note, however, that we likely underestimate the coastal protection function of the barrier, particularly for the first five kilometers west of the inlet. In this study, we do not account for wave setup and runup, which are effectively reduced on the back-barrier side of the spit system. While the latter two processes may have limited influence on water levels in Schleswig, it is well understood that the barrier breaks incoming waves, which would otherwise reach the inner coasts of the bay. Therefore, the contribution of the barrier to reducing peak water levels up to the area north of Kappeln is likely to be higher than the 13 cm presented in this study.

### The role of dikes for along-channel attenuation and coastal flooding along The Schlei

The low effectiveness of the investigated hypothetical MR sites in enhancing along-channel attenuation demonstrated in this study is in line with Smolders et al. (2015), who have analyzed along-estuary attenuation for a range of configurations of wetland elevations, locations and surface areas. While they found a high potential for large and low-lying wetlands to reduce peak water levels during spring tides (up to 0.2 m upstream), wetland locations toward the estuary mouth (also stressed by Fairchild et al. (2021)), higher wetland elevations and storm tides reduced water level differences to less than 0.03 m (Smolders et al., 2015). However, low-lying restoration sites may initially provide effective flood water storage, but are expected to silt up more rapidly, which can reduce the water storage capacity over time given sufficient sediments are available (Oosterlee et al., 2018, 2020; Liu et al., 2021).

The potential restoration sites along The Schlei are located near the inlet, are smaller in terms of surface area (a total of 373.65 ha across the three detected potential sites vs. between 1000 and 3000 ha) and higher in elevation compared to Smolders et al. (2015) (average elevation 2.42 m ± 2.67 m vs. 0.82 m, 1.4 m, 1.85 m and 2.50 m). For the latter elevation, water levels are even amplified along the Scheldt estuary by 13 cm as compared to a restoration site elevation of 1.85 m (Smolders et al., 2015). It may therefore not be surprising that our results suggest a limited influence of existing dikes along The Schlei on along-channel attenuation. Specifically, we found that peak water levels are reduced by 1 and 2 cm at observation point three when floodplains are restored by means of MR near The Schlei’s inlet (Figure 1).

The hypothetic MR sites along The Schlei increase the total flooding extent, as in our simulations large low-lying areas near Maasholm and west of observation point 3 were allowed to inundate in the model. Across the 2019 surge, SSP1-2.6 and SSP5-8.5 (medium), the increase in flooding extent due to MR varies only between 197.75 ha (SSP5-8.5 medium) and 224.75 ha (2019 surge), clarifying that it is mostly the MR area itself that leads to the increase in flooding extent (see also Table 1). For the highest SLR scenario, MR even results in a lower flooding extent as compared to status quo adaptation, which we ascribe to the associated increase in the new landward dike heights and the effects of the added bottom friction.

The hypothetic height increase for currently existing dikes by 1.5 m effectively reduces flooding extents near the inlet during the highest simulated SLR scenario. However, the overall contribution to flooding extent reduction along The Schlei is only 13% (Table 1 and Supplementary Figure S5). The reason is that the effectiveness of dikes in reducing flooding is limited to those locations where they are currently present, which is near the inlet up to observation point three (Figure 1).

## Conclusions

We conclude that fjord-like channels such as The Schlei can provide effective peak water level reductions, which we refer to as along-channel attenuation. However, this effect may be reduced when sea levels rise, potentially leading to a non-linear increase in peak water levels in the future. In The Schlei, most of the along-channel attenuation occurs at the narrow passages. However, meteorological conditions can substantially amplify peak water levels. The barrier spit system contributes to along-channel attenuation, as peak water levels in Schleswig during the 2019 surge are 9% higher when the barrier is removed (as compared to status quo). However, the importance of the barrier spit system for along-channel attenuation is reduced when sea levels rise.

Hypothetic changes to existing dikes along The Schlei in the form of height increases and MR are mostly influencing the flooding extent, while the effects on along-channel attenuation are small (<2 cm). We ascribe the limited influence of MR on along-channel attenuation to the small MR area as compared to the size of The Schlei’s water body, the location of MR near the inlet and relatively high elevations inside the potential MR sites. In summary, while previous studies have demonstrated the importance of MR for along-estuary attenuation, we conclude that currently limited potential areas for MR implementation lead to limited effects on along-channel attenuation inside The Schlei.

## Supporting information

Kiesel et al. supplementary material Kiesel et al. supplementary material

## Data Availability

The (peak) water levels for each observation point, hydrodynamic as well as landscape scenario, and the respective flooding extents are openly available from Kiesel et al. (2024).

## Supplementary material

The supplementary material for this article can be found at http://doi.org/10.1017/cft.2024.11.
