# Supplementary material for: The influence of wind and basin geometry on surge attenuation along a microtidal channel in the western Baltic Sea
Source: Camb Prism Coast Futur. 2024 Nov 25;2:e15. doi: 10.1017/cft.2024.11 (PMC12337574; doi:10.1017/cft.2024.11)
Supplement: Kiesel et al. supplementary material [file S2754720524000118sup001.pdf]

Supplementary Material to the article entitled

The influence of wind and basin geometry on surge attenuation along a microtidal channel in the western Baltic Sea

Joshua Kiesel[1,2*], Arne Knies[3], Athanasios T. Vafeidis[1]

[1]Department of Geography, Kiel University, 24118 Kiel, Germany

[2] Institute for Environmental Studies, Vrije Universiteit Amsterdam, Amsterdam, The Netherlands

[3]Institute of Geosciences, Kiel University, 24118, Kiel, Germany

*** Corresponding author:**
j.kiesel@vu.nl

1 Supplementary Figures and Tables

1.1 Supplementary Tables

Table S1: Summary of data used to setup the hydrodynamic model for The Schlei. This table was taken from Kiesel et al. (2023).

Dataset	Resolution	Source	Accuracy	Availability
Bathymetry	50 m	Federal Maritime and Hydrographic Agency (BSH)	NA	free, www.geoseaportal.de
Elevation	10 m	ATKIS® DGM 10 (LiDAR); State Office of Geoinformation, Surveying and Cadestre MV and State Office for Suveying and Geoinformation SH	0.5 - 2.0 m	
Elevation	1 m	ATKIS® DGM 1 (LiDAR); State Office of Geoinformation, Surveying and Cadestre MV and State Office for Suveying and Geoinformation SH	< 30 cm horizontally and 15 - 20 cm vertically in flat terrain	
Land cover	100 m	Corine (© European Union, Copernicus Land Monitoring Service 2018, European Environment Agency (EEA))	Geometric accuracy < 100 m; Thematic accuracy > 85 %	free, https://www.copernicus.eu/en

Dikes SH	shapefile	ATKIS®; State Office for Suveying and Geoinformation SH	The dataset contains full coverage of flood protection dikes in SH. We used the layers"rel01" and "geb03". Selected shapes are "Hochwasserdeich", "Hauptdeich", "Landesschutzdeich", "Überlaufdeich", "Leitdeich", "Schlafdeich", "Mitteldeich", "Binnendeich", "Hauptdeich 1. Deichlinie" and "2. Deichlinie".

Table S2: Reclassification scheme of Corine land cover classes ((© European Union, Copernicus Land Monitoring Service 2018, European Environment Agency (EEA)). This table was taken from Kiesel et al. (2023).

Corine land class	Model land class
continuous urban fabric	urban
discontinous urban fabric	urban
industrial or commercial units	urban
road and railway networks and associated land	traffic
port areas	urban
airports	urban
mineral extraction sites	urban
dump sites	urban
construction sites	urban
green urban areas	green urban areas
Sport and leasure facilities	green urban areas
non-irrigated arable land	agriculture
fruit tree and berry plantation	agriculture
pasture	agriculture
complex cultivation patterns	agriculture
land principally occupied by agriculture, with significant areas of natural vegetation	agriculture
borad-leaved forest	forest
coniferous forest	forest
mixed forest	forest
natural grassland	fallow/natural grasslands

moors and heathland	wetland
transitional woodland-shrub	fallow/natural grasslands
beaches, dunes, sand	unvegetated coastal sediment
sparseley vegetated areas	fallow/natural grasslands
inland marshes	wetland
peat bogs	wetland
saltmarshes	wetland
intertidal flats	unvegetated coastal sediment
water courses	Inland waterbodies/courses
water bodies	Inland waterbodies/courses
coastal lagoons	Inland waterbodies/courses
estuaries	Inland waterbodies/courses
sea and ocean	sea and ocean

Table S3: Different setups of Manning´s n coefficients taken from the literature and adapted from Kiesel et al. (2023). The coefficient in bold letters is the only one that changed between the setups moderate and moderate 2.

	Manning´s *n* coefficient			
Land Use Class	High	Low	Moderate	Moderate 2
Agriculture	0.06[1]	0.03[3,5]	0.04[1]	0.04[1]
Forest	0.2[1]	0.1[4,5]	0.15[3, 6, 7]	0.15[3, 6, 7]
Urban	0.15[1]	0.015 [6]	0.07[3]	0.07[3]
Wetland	0.08[1]	0.035[1]	0.06[1]	0.06[1]
Sea and ocean	0.03[2]	0.012[2]	0.02[2]	0.02[2]
Inland waterbodies/courses	0.06[2]	0.02[2]	0.035[2]	**0.02[2]**
Green urban areas	0.12[1]	0.035[1, 7]	0.07[1]	0.07[1]
Natural grasslands	0.042[1]	0.034[4]	0.035[7]	0.035[7]
Traffic	0.032[1]	0.013[5]	0.016[6, 7]	0.016[6, 7]
unvegetated coastal sediment	0.09[4]	0.03[1] / 0.025[6]	0.04[1, 3]	0.04[1, 3]
References	[1]Bunya et al., 2010; [2]Garzon and Ferreira 2016; [3]Wamsley et al., 2009; [4]Liu et al., 2013; [5]Papaioannou et al., 2018, [6]Hossain and Jia, 2009; [7]Dorn et al., 2014			

1.2 Supplementary Figures

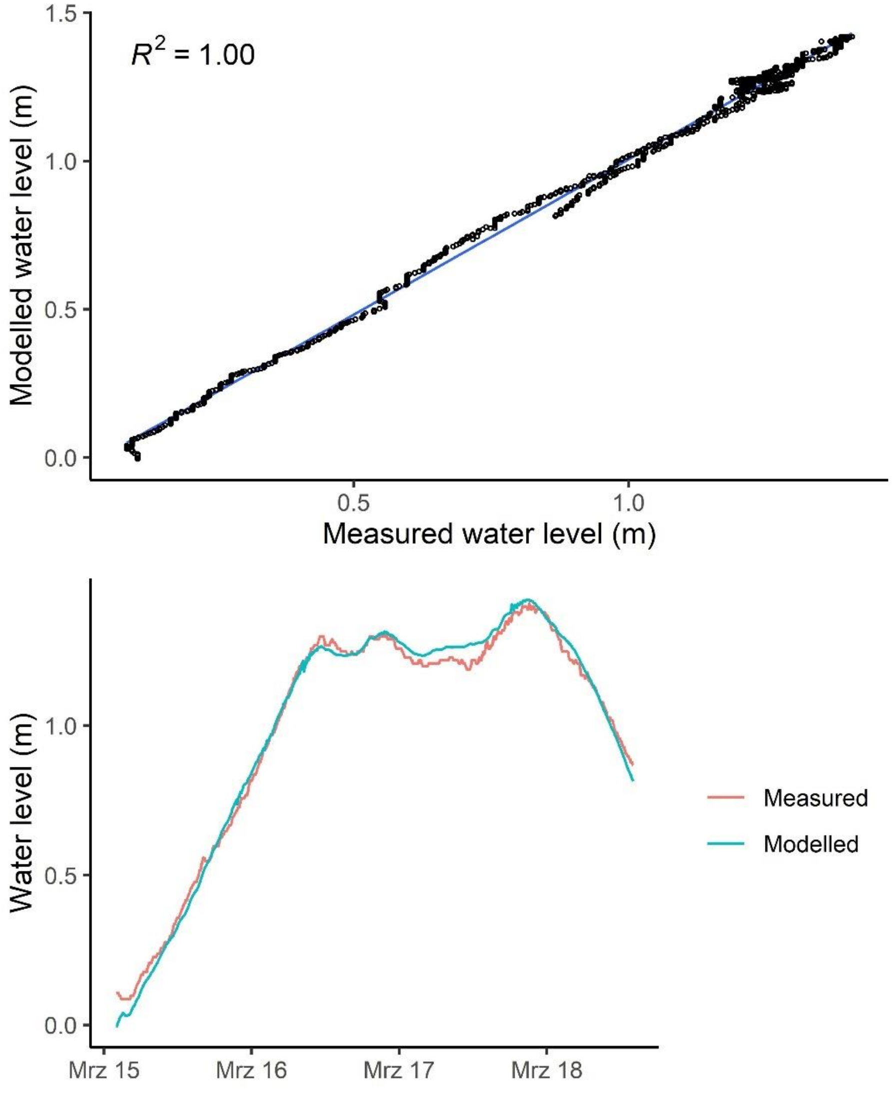

Figure S1: Results of the model validation. Top: Modelled vs. measured water levels at tide gauge Schleswig. Bottom: Timeseries of modelled and measured water levels in Schleswig.

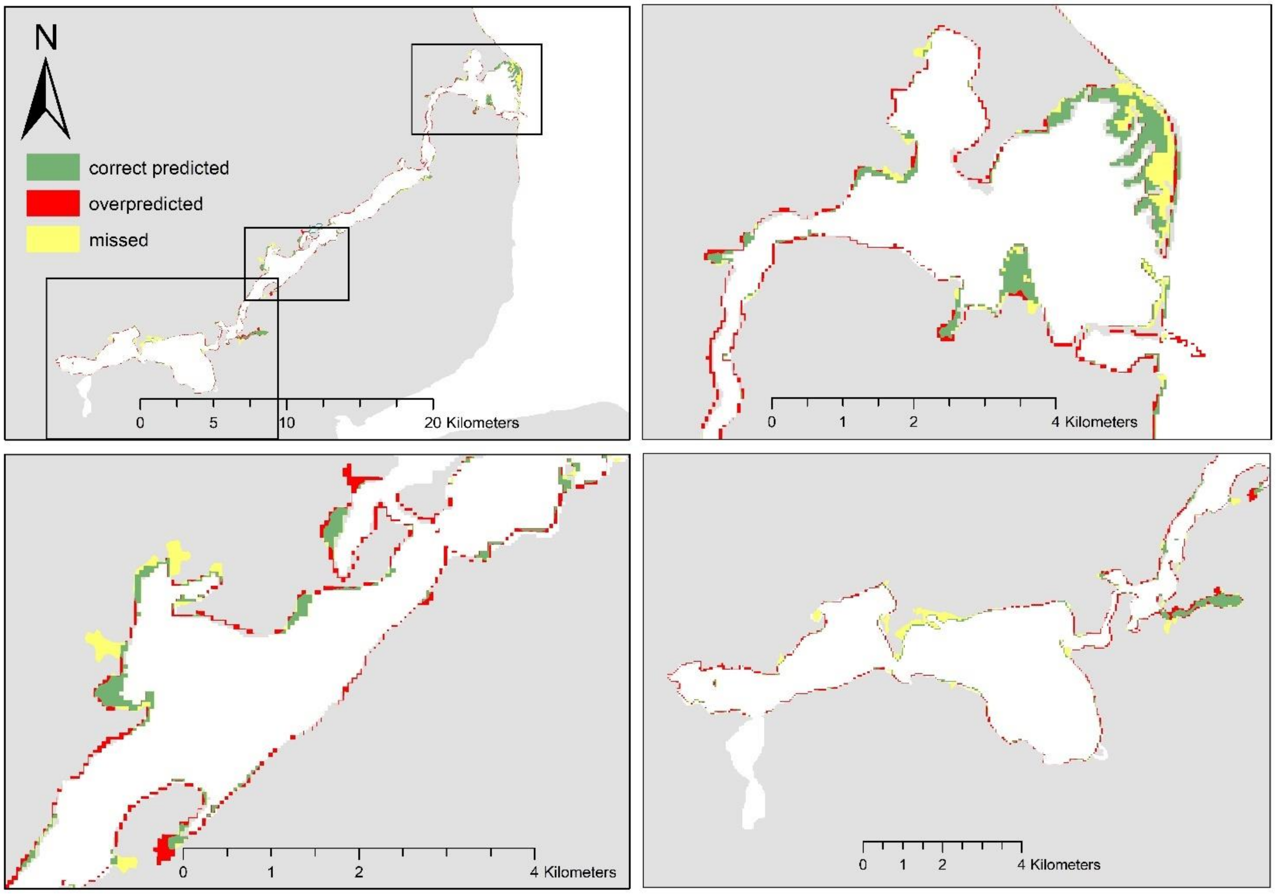

Figure S2: Comparison of flooding extents simulated with the hydrodynamic model and extracted from SAR-imagery for the storm surge of 2nd January 2019. We find that our model systematically overpredicts the flooding extent along the coastline. According to Kiesel et al. (2023), we ascribe this deviation to two main aspects; (1) the model resolution may not be fine enough to resolve the morphologically complex shoreline of The Schlei, featuring a mixture of wetlands such as saltmarsh and reed belts with extents often less than 50 m in width; (2) The suitability of using SAR imagery to map surface waters can be compromised if the contrast between water and adjacent (semi-)terrestrial areas is low. For instance, Mason et al. (2009) found that the accuracy of positioning the shoreline by means of SAR imagery was compromised by similar return signals of unflooded short vegetation and adjacent floodplains.

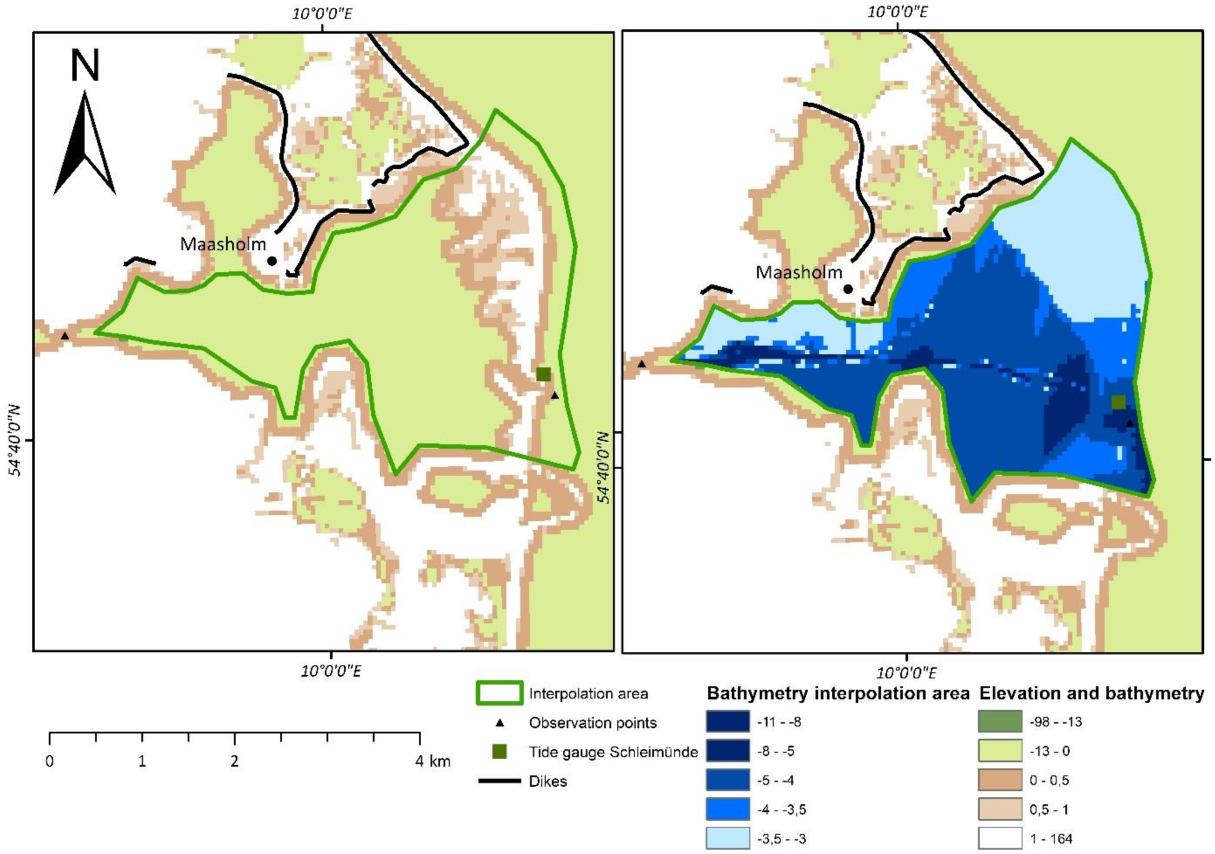

Figure S3: Elevation and bathymetry of The Schlei's inlet. Left: With sandspit and right, interpolated, counter-factual bathymetry without sandspit.

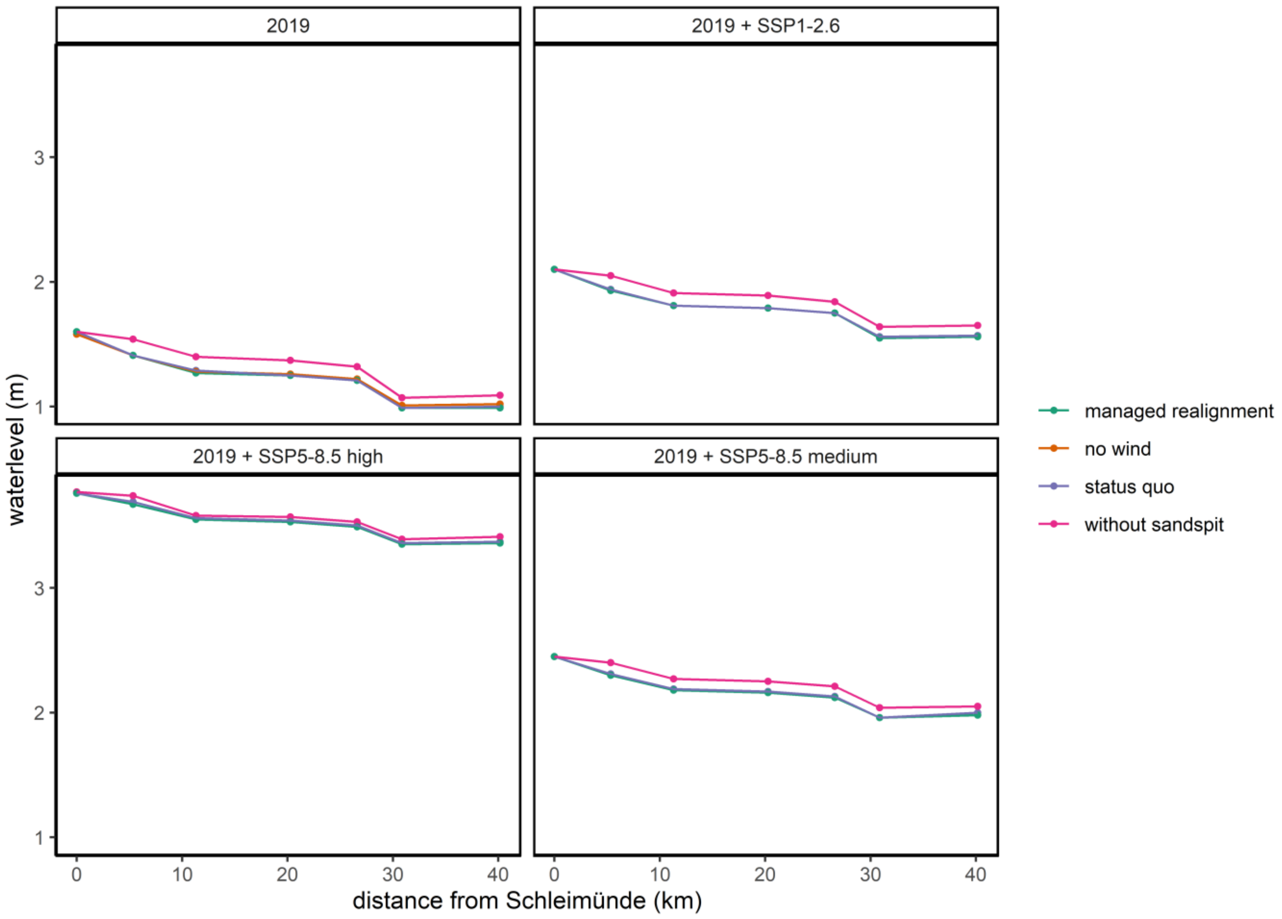

Figure S4: Along-channel attenuation for the storm surge from 2nd January 2019 and three sea-level rise scenarios. The effects of wind, the sandspit and managed realignment are indicated by different colours.

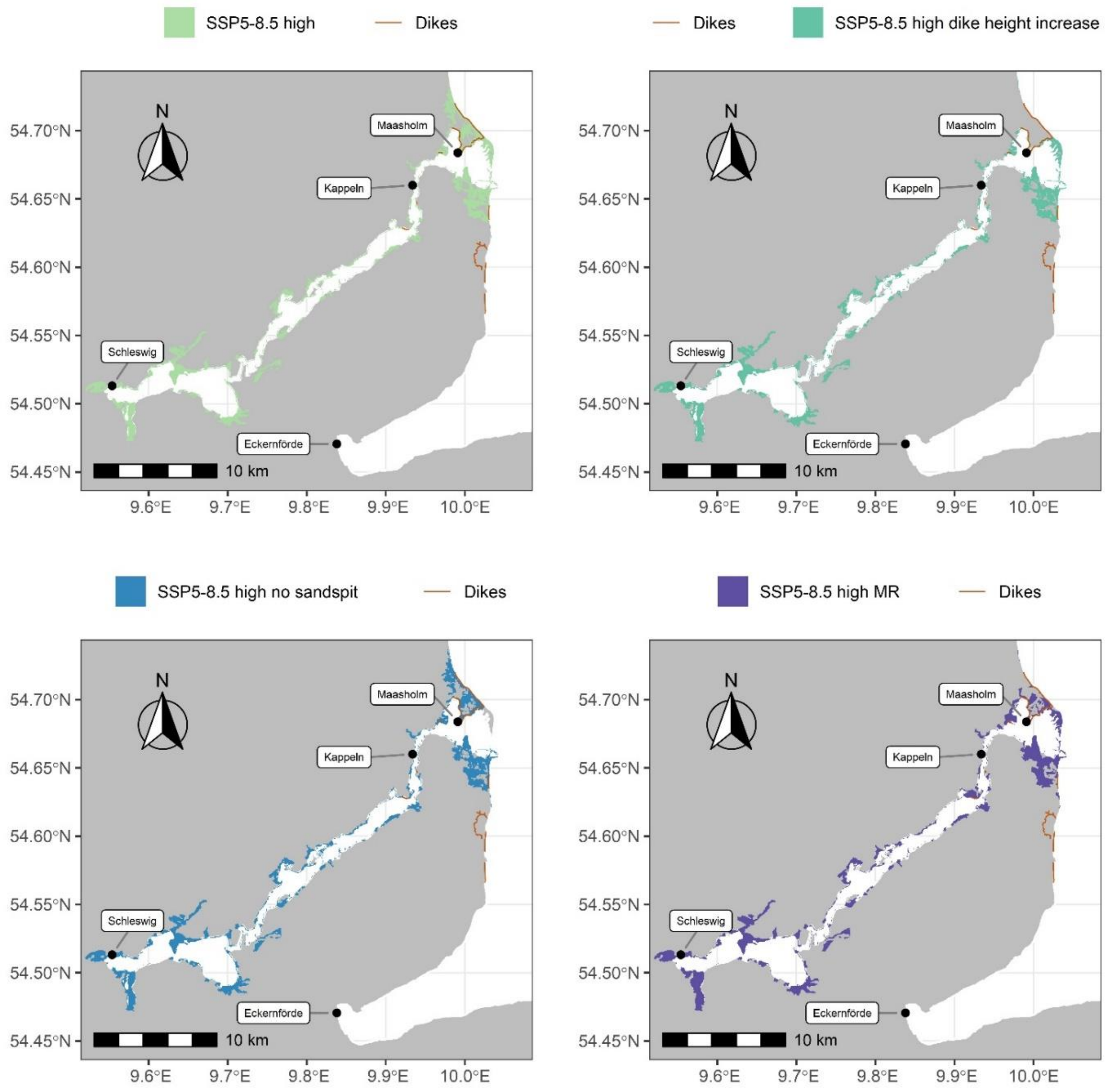

Figure S5: Flooding extents for SLR-scenario SSP5-8.5 high and the four landscape scenarios. Top left: Status quo. Top right: All dikes in the study region were increased in height by 1.5 m. Bottom left: The counter factual removal of the sandspit. Bottom right: Dikes were realigned where physically plausible.

2 Literature

Bunya, S., Dietrich, J. C., Westerink, J. J., Ebersole, B. A., Smith, J. M., Atkinson, J. H., Jensen, R., Resio, D. T., Luettich, R. A., Dawson, C., Cardone, V. J., Cox, A. T., Powell, M. D., Westerink, H. J., and Roberts, H. J.: A High-Resolution Coupled Riverine Flow, Tide, Wind, Wind Wave, and

Storm Surge Model for Southern Louisiana and Mississippi. Part I: Model Development and Validation, Mon. Weather Rev., 138, 345–377, https://doi.org/10.1175/2009MWR2906.1, 2010.

Dorn, H., Vetter, M., and Höfle, B.: GIS-Based Roughness Derivation for Flood Simulations: A Comparison of Orthophotos, LiDAR and Crowdsourced Geodata, Remote Sensing, 6, 1739–1759, https://doi.org/10.3390/rs6021739, 2014.

Garzon, J. and Ferreira, C.: Storm Surge Modeling in Large Estuaries: Sensitivity Analyses to Parameters and Physical Processes in the Chesapeake Bay, Journal of Marine Science and Engineering, 4, 45, https://doi.org/10.3390/jmse4030045, 2016.

Hossain, A., Jia, Y., and Chao, X.: Estimation of Manning's roughness coefficient distribution for hydrodynamic model using remotely sensed land cover features, in: 2009 17th International Conference on Geoinformatics, 1–4, IEEE, 12–14 August 2009, https://doi.org/10.1109/GEOINFORMATICS.2009.5293484, 2009.

Kiesel, J.; Lorenz, M.; König, M.; Gräwe, U.; Vafeidis, A. T. (2023): Regional assessment of extreme sea levels and associated coastal flooding along the German Baltic Sea coast. In: *Nat. Hazards Earth Syst. Sci.* (23), S. 2961–2985. DOI: 10.5194/nhess-23-2961-2023.

Liu, H., Zhang, K., Li, Y., and Xie, L.: Numerical study of the sensitivity of mangroves in reducing storm surge and flooding to hurricane characteristics in southern Florida, Cont. Shelf Res., 64, 51–65, https://doi.org/10.1016/j.csr.2013.05.015, 2013.

Mason, D., Bates, P., and Dall´Amico, J.: Calibration of uncertain flood inundation models using remotely sensed water levels, J. Hydrol., 368, 224–236, https://doi.org/10.1016/j.jhydrol.2009.02.034, 2009.

Papaioannou, G., Efstratiadis, A., Vasiliades, L., Loukas, A., Papalexiou, S., Koukouvinos, A., Tsoukalas, I., and Kossieris, P.: An Operational Method for Flood Directive Implementation in Ungauged Urban Areas, Hydrology, 5, 24, https://doi.org/10.3390/hydrology5020024, 2018.

Wamsley, T. V., Cialone, M. A., Smith, J. M., Ebersole, B. A., and Grzegorzewski, A. S.: Influence of landscape restoration and degradation on storm surge and waves in southern Louisiana, Nat. Hazards, 51, 207–224, https://doi.org/10.1007/s11069-009-9378-z, 2009.
