# Supplementary material for: The influence of wind and basin geometry on surge attenuation along a microtidal channel in the western Baltic Sea
Source: Camb Prism Coast Futur. 2024 Nov 25;2:e15. doi: 10.1017/cft.2024.11 (PMC12337574; doi:10.1017/cft.2024.11)
Supplement: Peer review history

# Editor report: Recommendation: The influence of wind and basin geometry on surge attenuation along a microtidal channel in the western Baltic Sea — R0/PR2

**DOI:** 10.1017/cft.2024.11.pr2

**Affiliations:** University of Cantabria, Spain

No accompanying comment.

# Editor report: Decision: The influence of wind and basin geometry on surge attenuation along a microtidal channel in the western Baltic Sea — R0/PR3

**DOI:** 10.1017/cft.2024.11.pr3

**Affiliations:** Geography, Cambridge University, United Kingdom of Great Britain and Northern Ireland

No accompanying comment.

# Editor report: Recommendation: The influence of wind and basin geometry on surge attenuation along a microtidal channel in the western Baltic Sea — R1/PR5

**DOI:** 10.1017/cft.2024.11.pr5

**Affiliations:** Wetlands and Aquatic Research Centre, USGS, United States

Associate Editor (Krauss): I enjoyed reading this paper and learning more about the wave/wind/surge dynamics of a system I have no experience working in. The manuscript is well-written, well-referenced, and the methods are acceptable. Revisions were acceptable as well. Thank you for submitting this manuscript to Coastal Futures.

# Editor report: Decision: The influence of wind and basin geometry on surge attenuation along a microtidal channel in the western Baltic Sea — R1/PR6

**DOI:** 10.1017/cft.2024.11.pr6

**Affiliations:** Geography, Cambridge University, United Kingdom of Great Britain and Northern Ireland

No accompanying comment.
